# Characterization, evolution and risk factors of diabetes and prediabetes in a pediatric cohort of renal and liver transplant recipients

**DOI:** 10.3389/fped.2023.1080905

**Published:** 2023-02-07

**Authors:** Sophie Welsch, Virginie Mailleux, Priscilla le Hardy de Beaulieu, Nadejda Ranguelov, Nathalie Godefroid, Annie Robert, Xavier Stephenne, Isabelle Scheers, Raymond Reding, Etienne M. Sokal, Philippe A. Lysy

**Affiliations:** ^1^ Pôle PEDI, Institut de Recherche Expérimentale et Clinique, UCLouvain, Brussels, Belgium; ^2^ Department of Pediatric Nephrology, Cliniques Universitaires Saint-Luc, Brussels, Belgium; ^3^ Pôle Epidémiologie et Biostatistique, Institut de Recherche Expérimentale et Clinique, UCLouvain, Brussels, Belgium; ^4^ Department of Pediatric Gastroenterology and Hepatology, Cliniques Universitaires Saint-Luc, Brussels, Belgium; ^5^ Department of Pediatric Surgery and Liver Transplantation, Cliniques Universitaires Saint-Luc, Brussels, Belgium; ^6^ Departement of Pediatric Endocrinology, Cliniques Universitaires Saint-Luc, Brussels, Belgium

**Keywords:** diabetes, hyperglycemia, impaired glucose tolerance, insulin resistance, liver transplantation, renal transplantation, glucocorticoids

## Abstract

**Background:**

Hyperglycemia (HG) and prediabetes are rarely sought in pediatric liver (LT) and renal (RT) transplantation, yet their presence indicates a high risk of diabetes and cardiovascular disease. The objectives of our DIABGRAFT study were to retrospectively (rDIABGRAFT) and longitudinally (pDIABGRAFT) characterize HG and (pre)diabetes in a cohort of children with LT or/and RT.

**Methods:**

We retrospectively analyzed risk factors of HG from 195 children with LT from 2012 to 2019 and twenty children with RT from 2005 to 2019 at Cliniques universitaires Saint-Luc. In addition, we prospectively followed four LT and four RT children to evaluate the evolution of their glucose metabolism.

**Results:**

Our rDIABGRAFT study showed that 25% and 35% of LT and RT children respectively presented transient HG and 20% of RT developed diabetes. The occurrence of HG was associated with the use of glucocorticoids and with acute events as graft rejection and infection. In our pDIABGRAFT cohort, biological markers of diabetes were in the normal range for HbA_1C_, fasting glucose and insulin levels. However, oral glucose tolerance test and glucose sensors showed insulin resistance, impaired glucose tolerance and HG in the post-prandial afternoon period.

**Conclusion:**

Our study shows that children with LT and RT were more at risk of developing HG when glucocorticoids were required and that HbA_1C_ and fasting glucose lack sensitivity for early detection of glucose intolerance. Also, measurement of glycemia immediately after the transplantation and in postprandial period is key to detect dysglycemia since insulin resistance prevailed in our cohort.

**ClinicalTrials.gov ID:**

NCT05464043.

## Introduction

Solid organ transplantation (SOT) is the therapeutic choice for patient in end-stage renal or liver disease. After transplant, immunosuppression is required to ensure graft survival but is associated with side effects, including glycemic disorders. One of the most frequent complications observed with immunosuppressants is hyperglycemia (HG), which increases the probability to develop prediabetes and overt diabetes. Prediabetes, an intermediate state between normal glucose homeostasis and overt diabetes, represents a major health problem because in 2012 it was estimated that 70% of the prediabetic American citizens (33.5%) will develop diabetes within their lifetime (1–3). Diabetes affects an ill-defined proportion of transplant patients (2%–53%) (4–7) and is common in the context of adult liver and renal transplantation (5, 8, 9). Yet the incidence of transient HG, and the progression to overt diabetes in pediatric liver and renal transplantation remain unknown. However, it is known that both are associated with an unfavorable acute prognosis (i.e., mortality, graft rejection, increased hospital stay) and an increased cardiovascular risk in the long term in adult patients, this risk being correlated to the presence of metabolic syndrome (9–12). In addition, the use of fasting blood glucose and HbA_1C_ levels might not allow early detection of impaired glucose tolerance (IGT) as a preamble to prediabetes. It is therefore essential to gather knowledge on the evolution of glucose in pediatric patients after SOT. The objectives of our DIABGRAFT study were to assess the incidence and associated risk factors of developing hyperglycemia in liver and renal transplant children and longitudinally analyze the evolution of glycemic profile (i.e., HG, IGT and diabetes) in these patients during the post-transplant period.

## Materials and methods

### Study design

The DIABGRAFT study was conducted in collaboration with the Pediatric Hepatology and Gastroenterology and Specialized Pediatrics (Endocrinology and Nephrology Units) Services of Cliniques universitaires Saint-Luc (CUSL) in Belgium (Brussels). This study was approved by the local ethical committee (CUSL and UCLouvain Hospital-Faculty Ethics Committee; approval number 2019/12MAR/118) and was conducted in accordance with the Declaration of Helsinki. Our study included liver and renal transplant pediatric patients (<18 years of age) at CUSL. Were excluded patients with a history of diabetes (i.e., type 1, type 2, neonatal or monogenic), pancreatitis, Down Syndrome, cystic fibrosis (*n* = 1), a second organ transplantation for our LT cohort (*n* = 4; cardiac, renal), patients deceased shortly after transplantation (<1 year, *n* = 14), and patients with incomplete medical record (*n* = 8).

**Figure 1:**
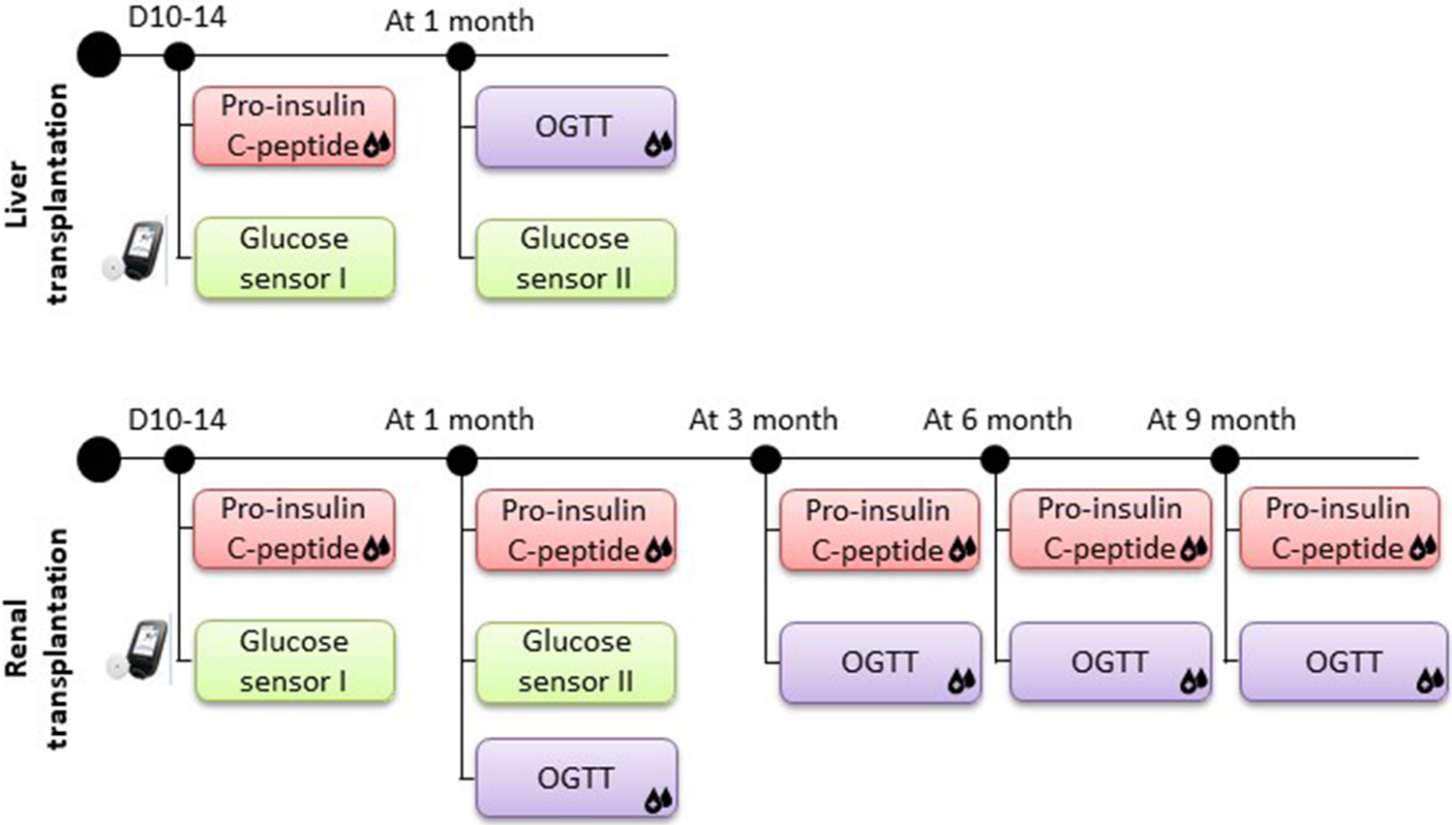
Protocol of prospective DIABGRAFT study. For LT and RT cohorts, pro-insulin and C-peptide secretion was measured after two weeks of transplant and measures had continued at one, three, six and nine months for RT patients. A glucose sensor was placed on the patient two weeks post-transplant for one month to detect the presence of early dysglycemia. An OGTT was performed after one month of transplantation and, for RT patients also at three, six and nine months.

DIABGRAFT was constituted of two parts. Its **retrospective part** (rDIABGRAFT) consisted of collecting data of pediatric patients who benefited from a liver transplant performed at CUSL between April 2012 and April 2019, or that benefited from a renal transplant in our center between September 2005 and April 2019. The **prospective part** (pDIABGRAFT) of the study consisted of a longitudinal glycemic evaluation of liver and renal transplant children in CUSL between 2020 and 2022 with the use of dynamic endocrine testing (Figure 1). Informed consents were collected from parents and from all children over six years of age.

### Treatments protocols for pediatric liver and renal transplant patients

At CUSL, liver and renal transplant children receive standard immunosuppression protocol as per international guidelines (13). For LT patients, this protocol includes the association of a monoclonal anti-CD 25 antibody (basiliximab, Simulect®) and a calcineurin inhibitor (tacrolimus, Prograft®) (14, 15). For RT patients, this protocol is based on a combination of Tacrolimus, glucocorticoids, monoclonal anti-CD 25 antibody and a cell proliferator inhibitor as mycophenolate mofetil (Cell-Cept®). Doses of glucocorticoids are introduced or increased when a LT/RT patient presents an acute cellular rejection (ACR). Complete treatment protocol is available in Supplementary data (Text S1).

About glucose monitoring, after a liver or renal transplantation at CUSL, glycemia is measured daily during hospitalization (between two weeks and one month) and for LT patients, glucose monitoring is regularly performed during a month until the patient returns to his home (after three months), after what yearly glycemic control is performed. For RT patients, the measure of fasting glycemia continues once weekly until the 6th month post-transplantation, when the control becomes once a month.

### Classification of glucose status

We defined hyperglycemia based on guidelines of the international consensus for diabetes of the American Diabetes Association (ADA): patients presented HG when fasting plasma glucose (FPG) or random plasma glucose (PG) levels exceeded respectively 126 mg/dl (7.0 mmol/L) and 200 mg/dl (11 mmol/L) for at least two measurements separated by 24 h, and not under a condition of stress such as the day of the transplant (16).

For our rDIABGRAFT study, the term “transient hyperglycemia” was used to define patients with HG (as described above) without overt diabetes diagnosed and diabetes was notified when patient required a persistent treatment (i.e., insulin or oral antidiabetics). For our pDIABGRAFT study, as we used dynamic testing, we classified our patient based on ADA guidelines: when a patient presented impaired fasting glucose (IFG) and/or impaired glucose tolerance (IGT) and/or HbA_1C_ from 5.7% to 6.4% (39–47 mmol/mol), we defined a “prediabetes” state. IFG was defined as FPG between 100 and 125 mg/dl (5.6 and 6.9 mmol/L) and IGT as 2h-PG levels during an oral glucose tolerance test (OGTT) from 140 to 199 mg/dl (7.8 and 11.0 mmol/L) (16). Diabetes was defined when a patient presented FPG ≥ 126 mg/dl (7.0 mmol/L) or a random PG or 2-h PG levels during OGTT > 200 mg/dl (11.1 mmol/L) or hemoglobin A1C (HbA_1C_) > 6.5% (48 mmol/mol) and/or when the patient presents classic symptoms of HG (16).

### Dynamic testing of glucose homeostasis

After obtaining the consent of pediatric patients and their parents, a glucose sensor (The FreeStyle Libre Flash Glucose Monitoring system, Abbott) was placed on the patient two weeks post-transplant for one month to detect the presence of early dysglycemia. Pro-insulin and C-peptide secretion by enzyme-linked immunosorbent assays (ELISA) was measured after two weeks of transplant to analyze the insulin secretion function of beta-cell, and measures had continued at one, three, six and nine months for RT patients. The enzyme-linked immunosorbent assays used for our analyses were performed as per manufacturer's instructions (Proinsulin 10-1118-01 and C-peptide 10-1136-01 kits, Mercodia). To analyze the insulin sensitivity and secretion over time, an OGTT was performed after one month of transplantation and, for RT patients also at three, six and nine months (Figure 1). Patients were not treated with insulin during tests. The OGTT was performed after 8 h overnight fast with weight-based glucose load (1.75 g/kg for pediatric patient) (17). Glucose and insulin were measured at fasting and at 30, 60 and 120 min after the ingestion of glucose. Insulin resistance (IR) was evaluated with HOMA-IR (for homeostasis model assessments of fasting insulin resistance; Ins^0^_(µU/ml)_ × Gluc(0)_(mmol/L)_/22.5) (18). If the HOMA index is less than 1.6, the result is normal. When the HOMA index is between 1.7 and 2.3, the patient presents a moderate form of IR and if the value is greater than 2.4, he suffers from a severe form of IR.

### Data collection

Patient history data included sex, date of birth, height, weight and gestation at birth (i.e., term, pre- or post-term), country of origin, date of death if patient deceased, the presence of hypo- and hyperglycemia in the neonatal period, dysmaturity, any chronic and hormonal treatment before the transplant, presence of dialysis for RT, its duration and type (e.g., hemodialysis, hemodiafiltration, peritoneal dialysis), endocrine or autoimmune diseases, acanthosis nigricans and sickle cell anemia. Also, we collected data about familial history as the presence or absence of consanguinity, metabolic syndrome, diabetes (type 1, type 2, gestational and monogenic), polycystic ovarian syndrome, fetal dystocia and sickle cell anemia.

We included information about the liver or renal transplant such as disease etiology, transplant date, the type of immunosuppressants administrated (tacrolimus, cyclosporine A, sirolimus, glucocorticoids), the use and duration of glucocorticoids in pre- and post-transplant period, the presence and date of liver or renal rejection, the type of transplant (living or cadaveric) and the link with the donor. We also collected anthropometric data in pre- and post-transplant period (at one, three, six and nine months after RT): weight, heigh, body max index (BMI) in standard deviation score (SDS) and Tanner stage. To obtain values in SDS score we used Belgian Flemish reference charts and Cole's Corpulence Curve (19, 20).

We collected glycemia and HbA_1C_ data before and after transplantation. When a patient presented HG after the first day of the transplantation, we reported the number of its occurrence, the date of its first and last observation and if a treatment was received (e.g., insulin therapy, antidiabetic oral), its doses per day, and its duration. The number of glycemia recorded was obtained by counting all measurements performed from the first consultation at CUSL (pre-transplantation evaluation) until the end of our data collection (in November 2021 for LT and in April 2022 for RT). The duration between the day of the transplantation and the last glycemia recorded was calculated to obtain the glycemia follow-up. We used REDCap (Research Electronic Data Capture) tool to collect and manage study data (21, 22).

### Statistical analysis

Discrete variables are described as numbers and percentages, and continuous variables were presented as medians with interquartile range (IQR). The characteristics of children were compared according to the occurrence or not of HG using Fisher exact test for discrete variables and Student t test or Mann-Whitney test for continuous variables. A binary logistic regression analysis was performed to predict HG occurrence from all potential predictors described in data collection section and results were expressed by estimating odds ratios (OR) with their 95% confidence intervals. Due to the low number of RT patients, only univariate analysis was performed. For our LT patients, covariates with a *p*-value less than 0.10 in univariate analysis were introduced into a multivariate model (Wald Chi-Square). The potential predictors “graft rejection” and “CMV (cytomegalovirus)” were not introduced in multivariate model due to their interaction with “glucocorticoids” and “infection” respectively. All *p*-values were two-sided/2-tailed and values less than 0.05 was considered statistically significant. All statistical analyses were performed with Stata® V17 software (Statacorp, Texas, USA).

## Results

### Patients and treatment characteristics of our rDIABGRAFT study

**Figure 2:**
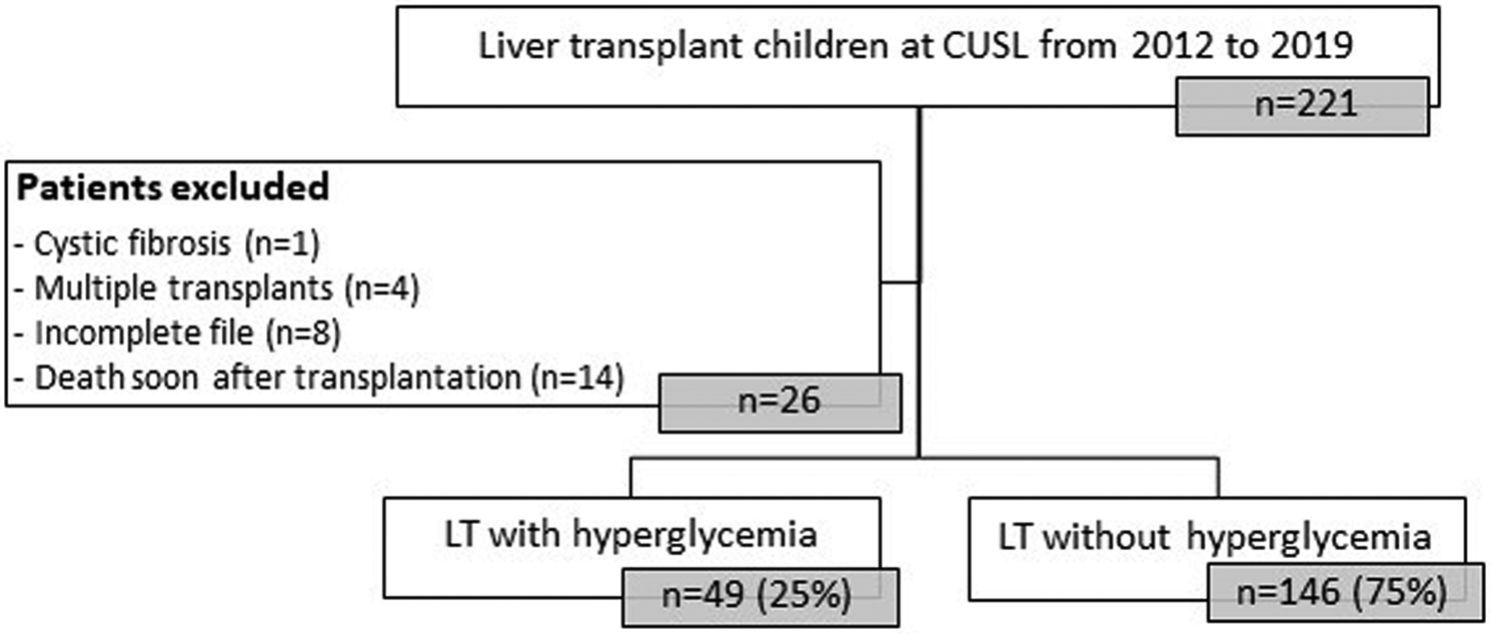
Flowchart of rDIABGRAFT pediatric LT cohort. Out of 195 pediatric patients who benefited from a liver transplant in the Cliniques universitaires Saint Luc (CUSL) between April 2012 and April 2019, 25% (49/195) patients presented hyperglycemia. n: number of patients, LT: liver transplant.

**Figure 3:**
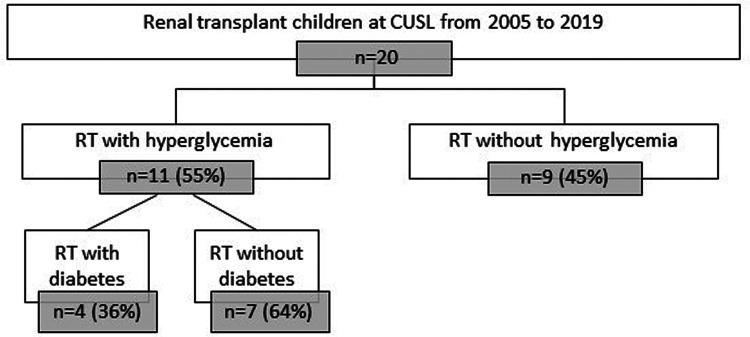
Flowchart of rDIABGRAFT pediatric RT cohort. Out of 20 pediatric patients who benefited from a renal transplant in the Cliniques universitaires Saint Luc (CUSL) between April 2004 and December 2019, eleven (55%) presented hyperglycemia. Out of them, four (20%) developed overt diabetes and the remaining seven (35%) patients presented HG without overt diabetes. n: number of patients, RT: renal transplant.

Characteristics of our rDIABGRAFT LT and RT cohorts are summarized in Tables 1, 2 (Supplementary Tables S1: LT countries and S2: LT pathologies), respectively. We collected data from 195 pediatric patients treated at CUSL with liver transplantation (LT) (Figure 2). The median age of liver recipients was 18 months (10; 36) and the majority (179/195) received a liver from a living donor. All patients were treated lifelong with tacrolimus and 65% (126/195) were temporally treated with glucocorticoids. Regarding acute complications, 44% (86/195) were diagnosed with a viral infection (nCMV = 55/195; nEBV = 42/195) and approximatively half (104/195) of our total LT cohort presented graft rejection, the majority of which was treated with glucocorticoids (91/104) whereas five patients (2.6%) required a second transplantation. About our renal transplantation (RT) cohort, we collected data about 20 pediatric patients (Figure 3). The median age of renal recipients was 12 years (9; 15), seven patients (35%) received a renal transplant from a living donor, all patients were treated with tacrolimus and glucocorticoids after the transplantation, and fourteen (70%) were still under both treatment at the end of data collection. For acute complications, fourteen (70%) presented infection: nine (45%) were diagnosed with a viral infection (nCMV = 4/20 and nEBV = 7/20) and seven (35%) presented bacterial infection. Seven (35%) patients presented a confirmed or borderline graft rejection for which they received shots or/and increased doses of glucocorticoids. Two patients (10%) were re-transplanted.

**Table 1:** Characteristic and treatment of pediatric liver transplant patients (rDIABGRAFT).

	LT total Cohort, *n* = 195	LT HG positive *n* = 49	LT HG negative *n* = 146
**CHARACTERISTIC**			
Gender, man, *n* (%)	98 (50,3)	24 (49.0)	74 (50.7)
Alive, *n* (%)	193 (99.0)	48 (98.0)	145 (99.3)
Age of liver transplant, months, median (p25; p75)	18.1 (10.1; 36.2)	11.9 (9.2; 22.4)	20.2 (10.5; 44,3)
Age ≤1 years (%)	72 (36.9)	25 (51.0)	47 (32.2)
Age ≤2 years (%)	122 (62.6)	38 (77.5)	84 (57.5)
Weight SDS, median (p25; p75)	−1.3 (−2.3; −0.4)	−1.5 (−2.7; −0.8)	−1.3 (−2.2; −0.3)
Height SDS, median (p25; p75)	−1.6 (−2.6; −0.6)	−1.8 (−2.6; −0.7)	−1.6 (−2.6; −0.6)
BMI SDS, median (p25; p75)	−0.9 (−1.7; +0.3)	−1.2 (−1.8; +0.1)	−0.8 (−1.7; +0.4)
**TRANSPLANTATION AND TREATMENTS**			
**Treatment before liver transplantation**			
Glucocorticoids, *n* (%)	21 (10.7)	6 (12.2)	15 (10.3)
Immunosuppressors, *n* (%)	8 (4.1)	2 (4.1)	6 (4.1)
**Living donor for liver transplant, *n* (%)**	179 (91.7)	44 (89.8)	135 (92.5)
Father	66 (33.8)	18 (36.7)	48 (32.9)
Mother	79 (40.5)	20 (40.8)	59 (40.4)
Aunt/Uncle	23 (11.8)	6 (12.2)	17 (11.6)
Siblings	2 (1.0)	–	2 (1.4)
Cousin	7 (3.6)	–	7 (4.8)
Grandparents	2 (1.0)	–	2 (1.4)
**Immunosuppressive treatments post-transplant**			
Tacrolimus, *n* (%)	195 (100.0)	49 (100.0)	146 (100.0)
Glucocorticoids, *n* (%)	126 (64.6)	39 (79.6)	87 (59.6)
Resumption of glucocorticoids	22 (11.3)	9 (18.4)	13 (8.9)
**COMPLICATIONS**			
**Acute graft rejection or suspicion**	104 (53.3)	36 (73.5)	68 (46.6)
** **Glucocorticoids doses elevation or treatment	91 (46.7)	33 (67.3)	58 (39.7)
**Viral infection post-transplant, *n* (%)**	86 (44.1)	30 (61.2)	56 (38.4)
** **CMV, *n* (%)	55 (28.2)	22 (44.9)	33 (22.6)
EBV, *n* (%)	42 (21.5)	10 (20.4)	32 (21.9)
** **Hepatitis C	4 (2.1)	1 (2.0)	3 (2.0)
**Second liver transplantation**	5 (2.6)	3 (6.1)	2 (1.4)

LT, Liver transplant; SDS, Standard deviation score; BMI, Body max index; PTLD, Post-Transplant Lymphoproliferative Disease; CMV, Cytomegalovirus; EBV, Epstein-Barr Virus.

**Table 2:** Characteristic and treatment of pediatric renal transplant patients (rDIABGRAFT).

	RT (*n* = 20)	RT HG positive (*n* = 11)	RT HG negative (*n* = 9)
**CHARACTERISTIC**			
Gender, man, *n* (%)	13 (65.0)	7 (63.6)	6 (66.7)
Alive, *n* (%)	19 (95.0)	10 (90.9)	9 (100.0)
Age of renal transplant, year, median (p25; p75)	12.3 (9.3; 15.6)	11.5 (10.4; 14.3)	13.1 (5.1; 16.5)
[0–8], *n* (%)	5 (25.0)	2 (18.2)	3 (33.3)
[9–18], *n* (%)	15 (75.0)	9 (81.8)	6 (66.7)
Overweight/Obesity before transplant, *n* (%)	4 (20.0)	3 (27.3)	1 (11.1)
Weight, SDS, median (p25; p75)	−0.9 (−2.0; −0.1)	−0.6 (−2.2; +0.0)	−1.1 (−1.9; −0.3)
Height, SDS, median (p25; p75)	−1.3 (−2.2; −0.6)	−2.1 (−2.5; −1.5)	−0.6 (−1.2; −0.4)
BMI, SDS, median (p25; p75)	−0.1 (−1.4; +0.7)	+0.3 (−1.0; +1.1)	−0.3 (−1.6; +0.2)
**PERSONAL HISTORY**			
**Other transplants (liver)**, *n* (%)	3 (15.0)	3 (27.3)	–
LT before RT, *n* (%)	2 (10.0)	2 (18.2)	–
LT the same day as RT, *n* (%)	3 (15.0)	3 (27.3)	–
**Treatment before renal transplantation**			
Glucocorticoids, *n* (%)	5 (25.0)	4 (36.4)	1 (11.1)
Immunosuppressors, *n* (%)	4 (20.0)	3 (27.3)	1 (11.1)
**Dialysis treatment**, *n* (%)	13 (65.0)	6 (54.5)	7 (77.8)
Hemodialysis, *n* (%)	10 (50.0)	6 (54.5)	4 (44.4)
Peritoneal dialysis, *n* (%)	9 (45.0)	5 (45.5)	4 (44.4)
**TRANSPLANTATION AND TREATMENTS**			
**Living donor for renal transplant, *n* (%)**	7 (35.0)	3 (27.3)	4 (44.4)
Father, *n* (%)	2 (10.0)	–	2 (22.2)
Mother, *n* (%)	3 (15.0)	1 (9.1)	2 (22.2)
Friend, *n* (%)	1 (5.0)	1 (9.1)	–
Sister, *n* (%)	1 (5.0)	1 (9.1)	–
**Immunosuppressive treatments post-transplant**			
Tacrolimus, *n* (%)	20 (100.0)	11 (100.0)	9 (100.0)
Glucocorticoids, *n* (%)	20 (100.0)	11 (100.0)	9 (100.0)
Patient treated until the end of the study, *n* (%)	14 (70.0)	7 (63.6)	7 (77.8)
**COMPLICATIONS**			
**Graft rejection or suspicion**	7 (35.0)	6 (54.5)	1 (11.1)
Graft rejection	3 (15.0)	3 (27.3)	–
Suspicion with immunosuppressors treatment	4 (20.0)	3 (27.3)	1 (11.1)
Glucocorticoids doses elevation or treatment	7 (35.0)	6 (54.5)	1 (11.1)
Tacrolimus doses elevated	1 (5.0)	1 (9.1)	–
**Infection post-transplant**, *n* (%)	14 (70.0)	10 (90.9)	4 (44.4)
Bacterial infection, *n* (%)	7 (35.0)	5 (45.5)	2 (22.2)
Virus infections, *n* (%)	9 (45.0)	6 (54.5)	3 (33.3)
CMV, *n* (%)	4 (20.0)	3 (27.2)	1 (11.1)
EBV, *n* (%)	7 (35.0)	5 (45.5)	2 (22.2)
**Weigh gain post-transplant**, *n* (%)	9 (45.0)	5 (45.5)	4 (44.4)
**Second renal transplant**	2 (10.0)	2 (18.2)	–

RT, Renal transplant; SDS, Standard deviation score; BMI, Body max index; LT, Liver transplant; PTLD, Post-Transplant Lymphoproliferative Disease; EBV, Epstein-Barr Virus; CMV, Cytomegalovirus.

### Early transient HG in pediatric LT patients is associated with glucocorticoid use, graft rejection and viral infection

**Figure 4:**
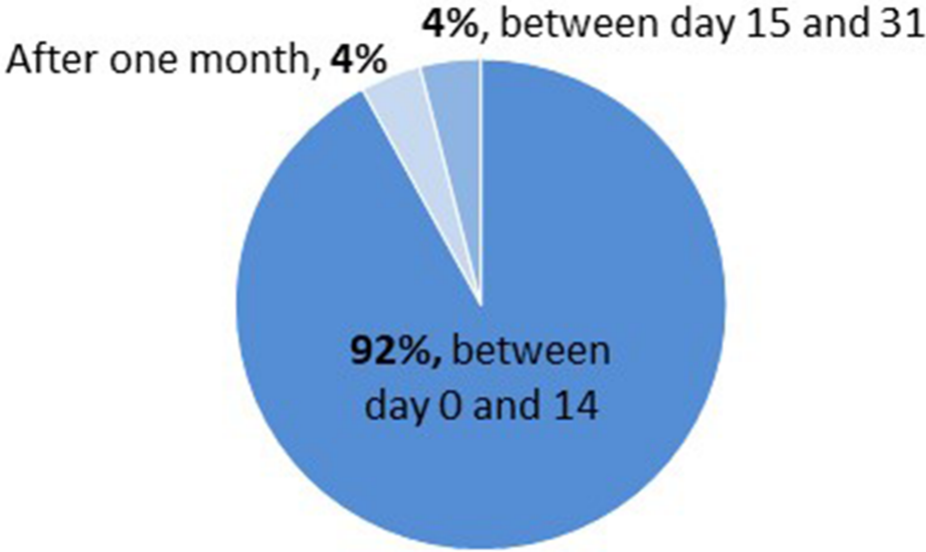
Onset of hyperglycemia in pediatric LT cohort (rDIABGRAFT). For most of our pediatric liver transplant patients (92%), hyperglycemia occurred during the first two weeks after transplantation.

Out of 195 LT pediatric patients, 25.1% (49/195) developed transient HG (Figure 2 and Table 3) and for most of them (92%) HG appeared during the first two weeks after transplantation (Figure 4 and Supplementary Figure S1). No overt diabetes was observed but a third (16/49) of our HG-positive LT cohort was treated with insulin.

**Table 3:** Incidence of hyperglycemia in LT cohort (rDIABGRAFT).

	Pediatric liver transplant patients, *n* = 195
Hyperglycemia the day of the transplantation, *n* (%)	115 (59.0)
Hyperglycemia, more than two days with glycemia >200 mg/dl, *n* (%)	49 (25.1)
Days in hyperglycemia, median (P25; P75)	4 (3; 8)
Transient insulin treatment, *n* (%)	16 (8.2)
Transient insulin treatment duration, days, median (P25; P75)	8 (2; 16)
Number of blood glucose levels recorded, median (P25; P75)	48 (37; 66)
Duration of blood glucose monitoring, days, median (P25; P75)	675 (325; 1355)

In univariate analysis, the use of glucocorticoids (OR 2.64 95% CI, 1.23–5.71) and the presence of critical condition such as graft rejection (OR 3.18 95% CI, 1.56–6.48) and viral infection (OR 2.54 95% CI, 1.31–4.93), in particularly Cytomegalovirus (OR 2.79 95% CI, 1.41–5.53) were significantly associated with the onset of HG as shown in Table 4. After adjustment with multivariate logistic regression analysis (Wald Chi-Square tests), incidence of transient HG after LT was higher in children who received glucocorticoids (2.96, 95% CI, 1.32–6.61) and presented a viral infection (OR 2.20, 95% CI, 1.09–4.44) (Table 4).

**Table 4:** Uni and multivariate analysis for LT cohort (rDIABGRAFT).

	LT HG positive	LT HG negative	Univariate analysis		Multivariate analysis	
	*n* = 49	*n* = 146	*p*-value	OR (95% CI)	*p*-value	OR (95% CI)
Glucocorticoids post-transplantation	39 (79.6)	87 (59.6)	0.01	2.64 (1.23–5.71)	0.01	2.96 (1.32–6.61)
Graft rejection	36 (73.5)	68 (46.6)	0.001	3.18 (1.56–6.48)	–	–
Virus infection	30 (61.2)	56 (38.4)	0.01	2.54 (1.31–4.93)	0.03	2.20 (1.09–4.44)
CMV	22 (44.9)	33 (22.6)	0.003	2.79 (1.41–5.53)	–	–

LT HG, Liver transplant hyperglycemia; OR, Odds ration; CI, Confidence interval; CMV, Cytomegalovirus.

### Pediatric LT patients present HG in afternoon, IR and diabetes at one-month

As we observed with rDIABGRAFT that our LT cohort presented transient HG early after transplantation (i.e., 0–14 days), we performed dynamic testing close to transplantation (day 14 and day 30) in four LT children. Table 5 presents the patients, treatments and characteristics of the pDIABGRAFT LT cohort.

**Table 5:** Pathology and glycemic profile data of LT cohort (pDIABGRAFT).

		TH1	TH2	TH3	TH4
Medical record	Genrer	Woman	Woman	Woman	Woman
	Country origin	Algeria	Algeria	Romania	Russia
	Pathology	Alagille Syndrome	Biliary cirrhosis, Progressive familial intrahepatic cholestasis	Budd-Chiari syndrome	Alagille Syndrome
	Donor	Living	Living	Living	Living
	Age of transplant, years	11.5	8.0	5.3	5.2
	Graft rejection	Yes	Yes	Yes	Yes
	Glucocorticoids	Yes	Yes	Yes	Yes
Secretion	[C-peptide], pmol/L	665.9	752.9	3142.0	1285
	[Pro-insulin], pmol/L	10.1	5.2	18.8	19.4
OGTT	Fasting glycemia, 0′	107	50	70	98
	Glycemia at 120′	122	212	–	250
	HOMA-IR	6.3	2.6	–	2.3

**Figure 5:**
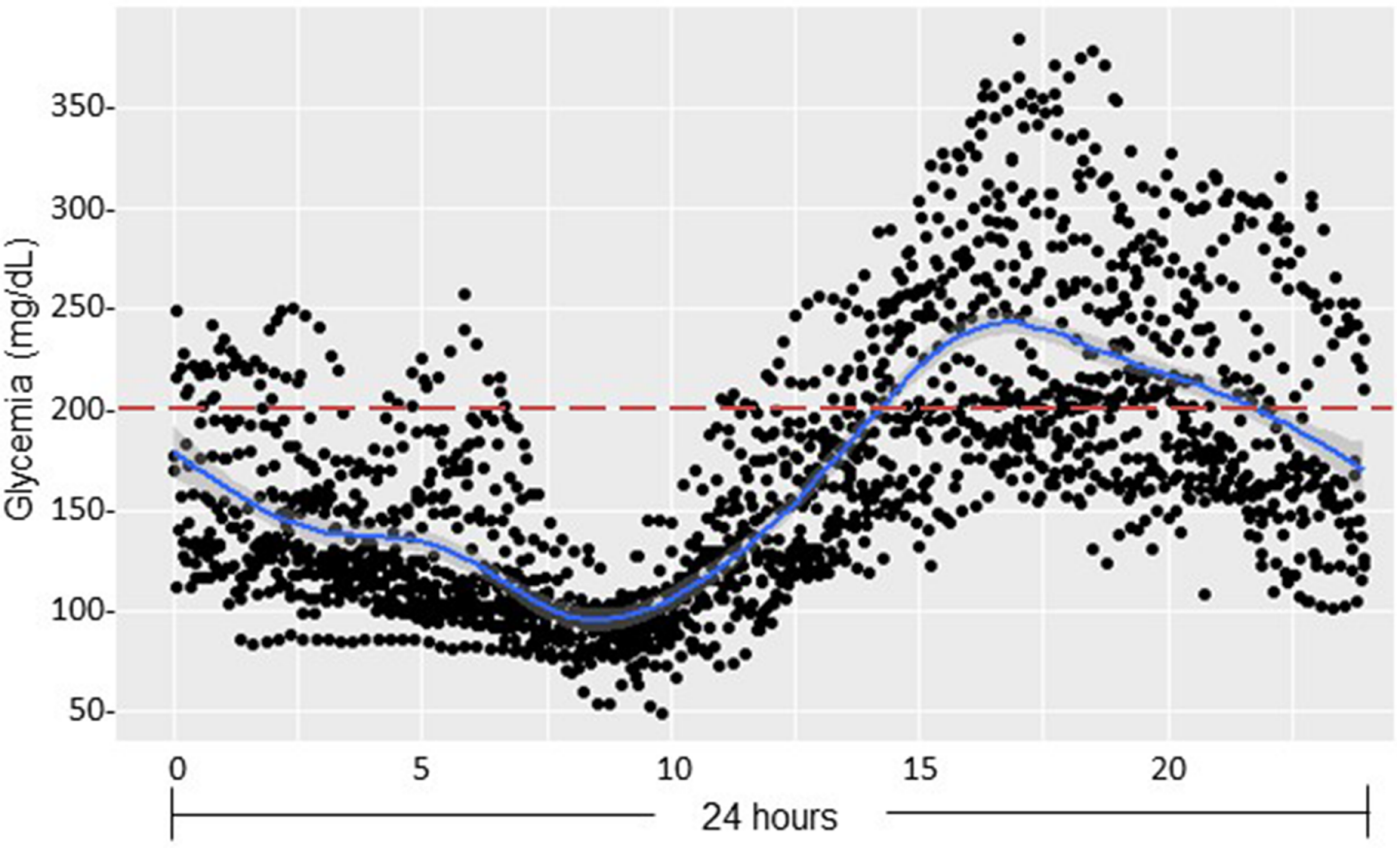
Continuous glucose monitoring after pediatric liver transplantation (pDIABGRAFT). Data of the continuous glucose monitor placed at day 14 post-LT for one month were regrouped on 24 h and showed chronic hyperglycemia occurring in postprandial afternoon period.

**Figure 6:**
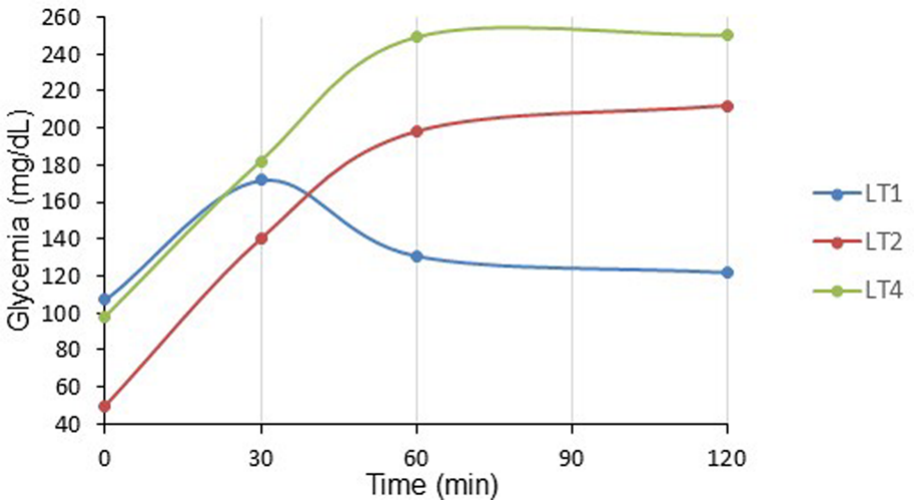
OGTT at one-month post liver transplant children (pDIABGRAFT). The oral glucose tolerance test (OGTT) performed at one-month post LT showed that fasting glucose were in the normal range whereas for two of them glycemia peaked respectively at 212 (LT2) and 250 (LT4) mg/dl at the end of the test (120′), corresponding to overt diabetes.

All patients presented fasting glucose and c-peptide level in normal range (Table 5) whereas glucose sensor placed at day 14 post-LT for one month showed chronic HG occurring in postprandial afternoon period (Figure 5). Parallelly, all children received high doses of glucocorticoids for graft rejection and required insulin. Moreover, during the OGTT performed at one-month post-LT (*n* = 3), all presented IR (HOMA-IR > 1.7) while in two of them, glycemia peaked respectively at 212 and 250 mg/dl at 120′ (Figure 6). We thus observed in our LT cohort two patients with diabetes at one-month post-LT.

### Chronic HG is associated with graft rejection and infection in pediatric RT patients

Out of our 20 pediatric patients with renal transplantation, 55% (11/20) presented HG (Table 6). Out of eleven patients with HG, four of them developed overt diabetes (20% of total cohort, 36% of HG cohort), still treated at the end of data collection with antidiabetic medication (oral antidiabetics in 2/4 and a combination of oral antidiabetics and insulin in 2/4). The remaining seven patients (35% of total cohort, 64% of HG cohort) presented HG without overt diabetes, during a median duration of seven days (6; 12) and four of them (57%) required insulin during a median duration of four days (2; 8).

**Table 6:** Incidence of hyperglycemia and overt diabetes in RT cohort (rDIABGRAFT).

	Pediatric renal transplant patients, *n* = 20
Glycemia >200 mg/dl the day of the transplantation, *n* (%)	10 (50.0)
**Hyperglycemia:** more than two days with glycemia >200 mg/dl	11 (50.0)
Transient hyperglycemia, *n* (%)	7 (35.0)
Days in hyperglycemia, median (P25; P75)	7 (6; 12)
Overt diabetes, *n* (%)	4 (20.0)
**Insulin and antidiabetic treatments,** *n* (%)	7 (35.0)
Insulin treatment for transient HG, *n* (%)	4 (20.0)
Duration of transient insulin treatment, day, median (P25; P75)	4 (2; 8)
Current antidiabetics treatment, *n* (%)	4 (20.0)
Current insulin treatment, *n* (%)	2 (10.0)
Total dose of insulin, Unit/kg/j, median (P25; P75)	0.30 (0.30; 0.32)
Number of blood glucose levels recorded, median (P25; P75)	136.5 (110; 229)
Years of blood glucose monitoring, median (P25; P75)	8.8 (6.3; 10.9)

No precise timing for developing HG was observed with our RT pediatric patients (Supplementary Figure S2), but a concomitance with the occurrence of critical events such as graft rejection and infection has been observed. Indeed, univariate analysis (Likelihood Ratio) was performed to evaluate the association between risk factors and HG, and our analysis showed that graft rejection (OR 14.0, 95% CI, 1.25–156.61) and infections post RT (OR 12.5 95% CI, 1.09–143.43) were significantly associated with a higher occurrence of HG (Table 7). All our patients with a re-transplantation or bi-organ transplantation (4/20; two second RT and two previous LT) presented chronic HG but logistic regression was not possible because no patient in the HG-free RT cohort required another transplant.

**Table 7:** Univariate analysis for RT cohort (rDIABGRAFT).

	RT HG positive	RT HG negative	Univariate analysis	
	*n* = 11	*n* = 9	*p*-value	OR (95% CI)
Graft rejection	7 (63.6)	1 (11.1)	0.03	14.0 (1.25–156.61)
Infection post-transplantation	10 (90.9)	4 (44.4)	0.02	12.5 (1.09–143.43)

RT HG, Renal transplant hyperglycemia; OR, Odds ration; CI, Confidence interval.

For our LT and RT pediatric patients, there was no difference between occurrence of HG and gender of patient, history of overweight/obesity, BMI (pre- and post-transplant) and the use of glucocorticoids before the transplantation, donor status (cadaveric or living), pathology requiring the transplant, weight gain or family history of diabetes.

### Insulin resistance and diabetes occur early after pediatric renal transplantation

For our RT cohort, as we did not observe a specific moment of HG occurrence but more a concomitance with the presence of critical events, we analyzed the evolution of glucose over time (at one-, three-, six- and nine-months post RT). Then, we followed four RT pediatric patients (Table 8). One RT patient (TR1) disagreed to use glucose sensor at one month post RT.

**Table 8:** Glycemic profile data of RT cohort (pDIABGRAFT).

			TR1	TR2	TR3	TR4
Medical record	Genrer		Man	Man	Man	Woman
	Country origin		Belgium	Romania	Romania	Belgium
	Donor		Cadaveric	Cadaveric	Cadaveric	Cadaveric
	Age of transplant, year		5.1	6.8	16.8	14.9

	Patients	14 days	1-month	3-month	6-month	9-month
C-peptide (pmol/L)	TR1	899	608	317	392	368
	TR2	748	798	344	1887	444
	TR3	1891	1918	1075	1101	848
	TR4	1122	3832	725	541	571
Pro-insulin (pmol/L)	TR1	11.7	4.5	3.9	2.5	2.1
	TR2	9.4	13.0	4.6	27.8	5.2
	TR3	19.0	19.5	9.5	12.2	7.2
	TR4	11.8	48.0	4.3	4.0	4.2
HbA1c	TR1		5.5	4.9	6.0	5.3
	TR2		-	5.8	5.6	5.4
	TR3		5.2	5.1	5.7	5.9
	TR4		5.1	5.0	4.9	5.4
Fasting glucose (OGTT 0′)	TR1		84	91	–	82
	TR2		88	87	84	93
	TR3		98	100	96	84
	TR4		76	80	88	85
OGTT 120′	TR1		221	120	–	81
	TR2		141	126	105	122
	TR3		120	130	113	109
	TR4		172	97	103	125
HOMA INDEX	TR1		1.7	2.1	–	1.2
	TR2		2.8	3.0	2.7	2.8
	TR3		3.7	3.6	5.1	1.7
	TR4		3.0	3.5	3.6	4.3

**Figure 7:**
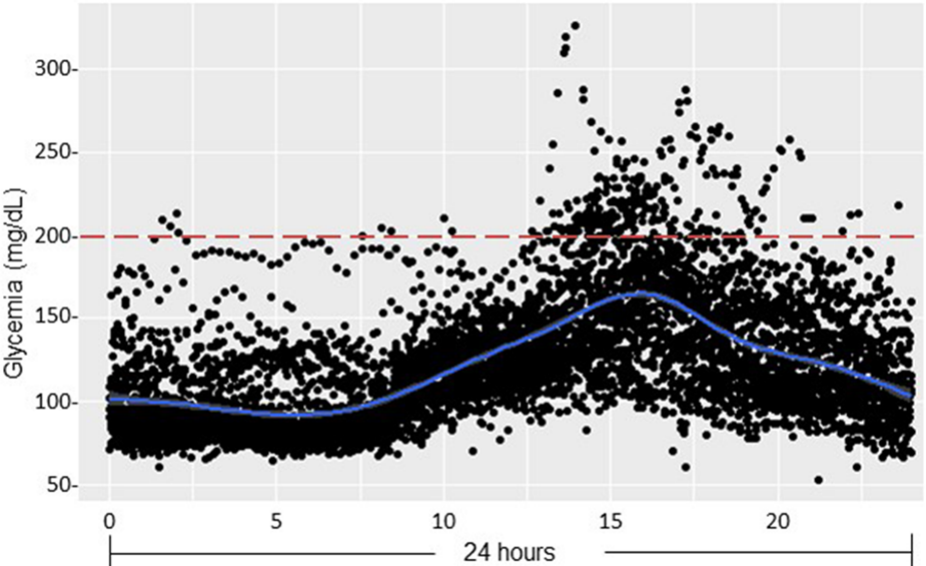
Glucose sensor data after pediatric renal transplantation (pDIABGRAFT). Data of the glucose sensor placed at day 14 post-RT for one month were regrouped on 24 h and showed hyperglycemia in postprandial afternoon period.

**Figure 8:**
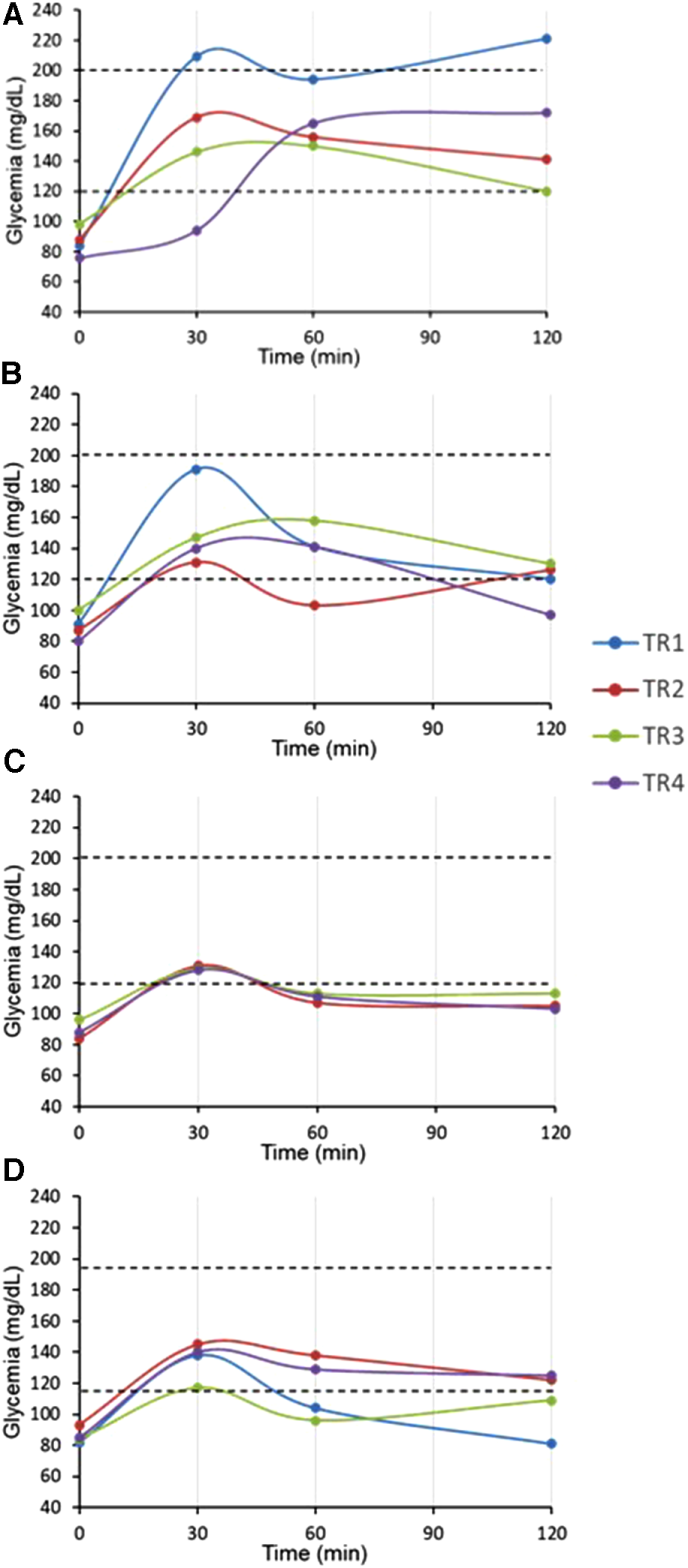
OGTT at one, three, six and nine-month post-renal transplant children (pDIABGRAFT). The oral glucose tolerance test (OGTT) performed at (**A**) one-month post RT showed that fasting glucose were in the normal range whereas two patients (TR2, TR4) presented impaired glucose tolerance (>140 mg/dl) at the end of the test, suggesting prediabetes and one presented glycemia above 200 mg/dl (TR1) at 120′, corresponding to overt diabetes. At (**B**) three-, (**C**) six- and (**D**) nine-months post-RT, all patients had normalized their glycemia at the end of the test.

Our analyses showed that all our RT pediatric patients presented normal fasting glycemia and HbA_1C_ levels from all the post-transplant follow-up period (up to 9 months) (Table 8). Moreover, glucose sensor (placed after 2 weeks post-RT) data showed HG in the afternoon as illustrated in Figure 7, with data regrouped on 24 h. OGTT performed at one-month post RT showed that two patients presented IGT with glycemia above 140 mg/dl at the end of the test, suggesting prediabetes, and one presented glycemia above 200 mg/dl (TR1: 221 mg/dl) at 120′, corresponding to overt diabetes (Figure 8). Dosage of pro-insulin and C-peptide showed that no patient presented a β-cell dysfunction whereas HOMA-IR showed severe IR (HOMA-IR >2.4) for all our RT patients (Table 8). At three-, six- and nine-months post-RT, all patients had normalized their glycemia at the end of the test (<140 mg/dl) (Figure 8), but they had continued to present moderate and severe IR except one at nine-month (Table 8).

## Discussion

Our study describes the incidence and risk factors of hyperglycemia and analyzes glycemic profile in a cohort of liver and/or renal pediatric transplant patients. To our knowledge, our study is the only one that combines retro- and prospective parts which include glucose screening test rarely performed in pediatric patients who benefited from a liver or kidney transplant.

In our LT cohort, 25% (49/195) of pediatric patients presented early HG with no overt diabetes afterwards. For our pediatric RT cohort, 55% (11/20) of pediatric patients presented HG. For 35% of them (7/20), HG were transient and the remaining 20% (4/20) developed overt diabetes, currently treated with antidiabetic treatment (insulin and/or oral antidiabetics). Studies performed before 2014 were based on variable definitions of diabetes, but the introduction of recommendation in 2014 by the American Journal of Transplantation and guidelines in 2017 by ADA for “post-transplantation diabetes” induced the observation of rates of diabetes closer to our results (23, 24). Indeed, the recent study by Calani et al. reported 13% (17/127) of diabetes in RT pediatric patients (25).

In a prevention perspective, we sought to identify relevant risk factors of HG onset after a pediatric LT and RT. The first finding of our DIABGRAFT study was, as expected, the association between HG and the use of glucocorticoids for LT cohort. The negative effect of glucocorticoids on glucose metabolism is well documented in transplant children (26–28). Associated to the use of glucocorticoids, graft rejection was also correlated to the risk of HG in our univariate analysis for our both cohorts. According to the immunosuppressive treatment protocol, high doses of glucocorticoids are introduced for LT and increased for RT when a patient presents ACR (14). The other risk factor of HG observed for our both cohorts was the presence of infections and can be explained by two hypotheses. Various studies described that following a metabolic stress such as infection in this case, various hormones such as cortisol, glucagon, catecholamines and pro-inflammatory cytokines are secreted and may provoke HG onset (29–32). In parallel, HG concomitant to an infection also may be related to an intensive prior immunosuppressive treatment (33). Our study suggests that these three risk factors of HG indicated a specific moment when a LT and RT patient has a higher risk of developing HG, when glucocorticoids were required and when a graft rejection and an infection occur.

We did not observe risk factors as older age at the time of the transplant and history of overweight/obesity usually seen in adults (5, 7, 12, 34), potentially because our cohorts were principally composed by liver transplant patients under the age of two years and underweighted. Also, overweight/obese patients waiting for a kidney transplant were on a specific diet to lose weight before transplantation.

In addition, the high proportion of transient HG and overt diabetes observed in our RT cohort compared to our LT cohort can be explained by several hypotheses. RT patients were directly administrated glucocorticoids for at least six months after transplantation, although LT patients received this treatment only in some specific cases, as graft rejection (15, 35, 36). In addition, our RT patients were pubertal (12 years, Tanner stage ≥2), whereas the majority of LT cohort was under the age of two (Tanner stage = 1) and in agreement with our previous study, with pediatric patients treated with glucocorticoids for a leukemia, Tanner stage ≥2 is associated with a higher risk of developing HG (37).

The other main finding of our DIABGRAFT study was that pediatric LT and RT patients developed early IGT and IR after the transplant. In our study, the normal C-peptide levels secretion showed that there was no effect of glucocorticoids or tacrolimus on β cell function, but the globally abnormal values of OGTTs showed that all our transplant patients developed IGT by the installation of IR already at one-month post-transplant, until 9-month for our RT cohort. In addition, our glucose sensor and OGTT data confirmed that non-fasting glucose monitoring (i.e., random) should be widely recommended for early detection of glucose abnormalities and that fasting plasma glucose and HbA1C measurements lack power/sensibility to identify post-prandial hyperglycemia. Indeed, in our both cohorts, all pediatric transplant patients had fasting blood glucose and HbA_1C_ in the normal range whereas glucose sensor confirmed the presence of HG in post-prandial afternoon period and values of OGTT indicated the presence of prediabetes and the onset of diabetes. Our findings are similar to a recent study carried out on Egyptian pediatric kidney transplant recipients where OGTT was able to detect a high proportion of abnormalities in glucose metabolism (23.3%) (38). The increase of glycemia in post-prandial afternoon period is widely described and related to the use of glucocorticoids. Studies characterizing the circadian glycemic pattern by Burt et al. showed that the glucose peak after 8 h of the prednisolone administration corresponds to the action peak of prednisolone (39, 40).

Our study presented some limitations. First, the retrospective nature was a limitation although we excluded patients with an incomplete medical record. In addition, like for any surgical intervention, clinical parameters, including glycemia, are frequently recorded close to the surgery and less afterwards. Moreover, it may be expected that patients with a critical condition such as graft rejection and infection had benefited from a closer control of glycemia included in the global clinical parameters compared to patient without complication. Also, we highlighted with our prospective study that HG appeared in the post-prandial afternoon period whereas in our retrospective study, glycemia collected in patient medical record was often carried out in the fasting stage due to the tacrolimus dosing protocol. Thus, we obtained a potential underestimation of the occurrence of HG. Finally, since CUSL is an international center for pediatric liver transplantation, our patients and their parents were mostly foreigners and recruitment could be less effective even with the intervention of a translator. In parallel, since patients were returning home after surgery, the monitoring of glycemia by our center was performed every six months then annually.

In conclusion, diabetes is a major side effects in RT children (20%) and transient HG are frequent after a pediatric liver (25%) and renal (35%) transplant yet underestimated due to fasting glycemic measures and HbA_1C_. The onset of HG systematically occurred in the post-prandial afternoon period and was associated to the use of glucocorticoids and with acute events as graft rejection and infection. HG was characterized by IGT and IR early after transplantation, and only detected by OGTT. Our study suggests that random blood glucose monitoring should be reinforced in the afternoon period when children present critical complications such as graft rejection and infections.

## Data Availability

The original contributions presented in the study are included in the article/**Supplementary Material**, further inquiries can be directed to the corresponding author/s.
