# Supplementary material for: Characterization, evolution and risk factors of diabetes and prediabetes in a pediatric cohort of renal and liver transplant recipients
Source: Front Pediatr. 2023 Feb 7;11:1080905. doi: 10.3389/fped.2023.1080905 (PMC9941739; doi:10.3389/fped.2023.1080905)
Supplement: Supplementary file 1 [file Datasheet1.pdf]

Supplementary Material

Text S1: Treatment protocols for pediatric liver and renal transplant patients.

Table S1: Countries of pediatric liver transplant patients.

Table S2: Pathologies of pediatric liver transplant patients.

Figure S1: Visualization of blood glucose levels in pediatric liver transplant patients. Graph shows glycemia data of liver transplant children over a period of one year.

Figure S2: Visualization of blood glucose levels of pediatric renal transplant patients. Graph shows glycemia data of renal transplant children over a period of one year.

Text S1. Treatment protocols for pediatric liver and renal transplant patients.

At CUSL, liver and renal transplanted children receive standard immunosuppression protocol as per international guidelines[1]. For LT patients, this protocol includes the association of a monoclonal anti-CD 25 antibody (basiliximab, Simulect®) and a calcineurin inhibitor (tacrolimus, Prograft®)[2,3]. For RT patients, this protocol is based on a combination of Tacrolimus, glucocorticoids, Simulect and a cell proliferator inhibitor as mycophenolate mofetil (Cell-Cept®).

Oral administration of tacrolimus is high during the first two months after the transplantation for LT cohort (blood levels target at 8-10 ng/mL) and during the first three weeks for RT cohort (blood levels target at 10-12 ng/mL) and gradually decreases (LT: 6-8 ng/mL the third month and 4–6 ng/mL between three months and one year; RT: 8-10 ng/mL between D22 and D60 and 5–8 ng/mL after sixty days) until a lifelong maintenance dose depending on the patient clinical evolution (LT:1-3 ng/mL; RT: 4-6 ng/mL after six months). For LT patients, steroids (Solumedrol®, Medrol®) are administrated when they present an acute cellular rejection (ACR), approximatively between the 7th and the 14th day post-transplant. Patients receive high doses of steroids (5 mg/kg/day) which progressively decrease until the third month after rejection (0.25 mg/kg/day) and space out in the sixth month (0.5 mg/kg/2 days)[2]. For RT patients, high doses of Solumedrol (125mg/m^2) are administrated intravenously during surgery and on day one. Then, oral administration of prednisone begins at day two until day four at a high dose (60mg/m^2/j) and gradually decreases (10mg/m^2 in the second month and 5mg/m^2 in the third month) until the sixth month where its necessity is evaluated. Doses of glucocorticoids are increased when a LT and RT patients presents ACR.

References

1. Agency EM. Guideline on clinical investigation of immunosuppressants for solid organ transplantation. *Committee for medicinal products for human use (CHMP)* 2008;Doc. Ref. CHMP/EWP/263148/06.
2. de Magnée C, Brunée L, Tambucci R, et al. Is ABO-Incompatible Living Donor Liver Transplantation Really a Good Alternative for Pediatric Recipients? *Children (Basel).* 2021;8(7).
3. Gras JM, Gerkens S, Beguin C, et al. Steroid-free, tacrolimus-basiliximab immunosuppression in pediatric liver transplantation: clinical and pharmacoeconomic study in 50 children. *Liver Transpl.* 2008;14(4):469-477.

Table S1. Countries of pediatric liver transplant patients (rDIABGRAFT).

**Country**	**Number of patients, n=195**
**Africa**, n=88	
Algeria	58
Israel	19
Morocco	7
Tunisia	2
Guinea	1
Syria	1
**Eastern Europe**, n=63	
Ukraine	28
Russia	26
Romania	5
Moldova	2
Poland	2
**Western Europe**, n=32	
Belgium	28
France	2
Luxemburg	1
Netherlands	1
United Kingdom	1
**Southern Europa**, n=7	
Greece	4
Italia	2
Portugal	1
**Asia**, n=4	
Uzbekistan	2
India	1
Kazakhstan	1

Table S2. Pathologies of pediatric liver transplanted patients (rDIABGRAFT)

**Country**	**Number of patients, n=195**
**Cholestatic disease**, n=144	
Bile duct astresia	118
Alagille syndrome	12
Familial progressive intrahepatic cholestasis	10
Sclerosing cholangitis	3
Cholestasis	1
**Metabolic and genetic liver disease**, n=25	
Tyrosinemia	10
Crigler-Najjar	5
Alpha 1 antitrypsin deficiency	1
Carbamoyl phosphate synthetase deficiency	1
Ornithine carbamoyl transferase deficiency	1
Glucuronyl transferase deficiency	1
Familial hypercholesterolemia	1
Glycogenosis	1
Maple syrup urine, leucinose	1
Wilson disease	1
Nephronophthisis	1
Zellweger syndrome	1
**Malignant liver disease**, n=12	
Hepatoblastoma	10
Hepatocarcinoma	2
**Chronic liver disease**, n=10	
Cirrhosis of unknown origin	6
Autoimmune liver disease	3
Congenital hepatic fibrosis	1
**Acute liver failure**, n=4	
Toxic-allergic hepatitis	2
Neonatal herpic hepatitis	1
Budd Chiari	1

Figure S1

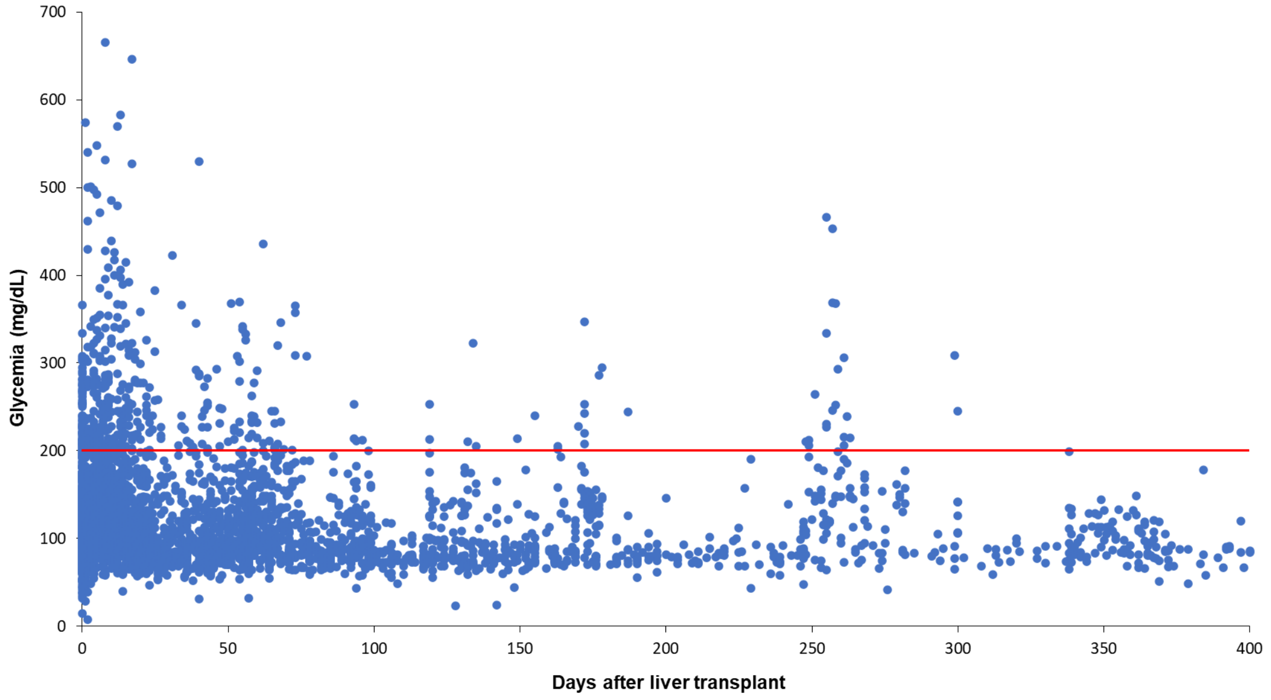

700
600
500
400
300
200
100
0
Glycemia (mg/dL)
0
50
100
150
200
250
300
350
400
Days after liver transplant

Figure S2

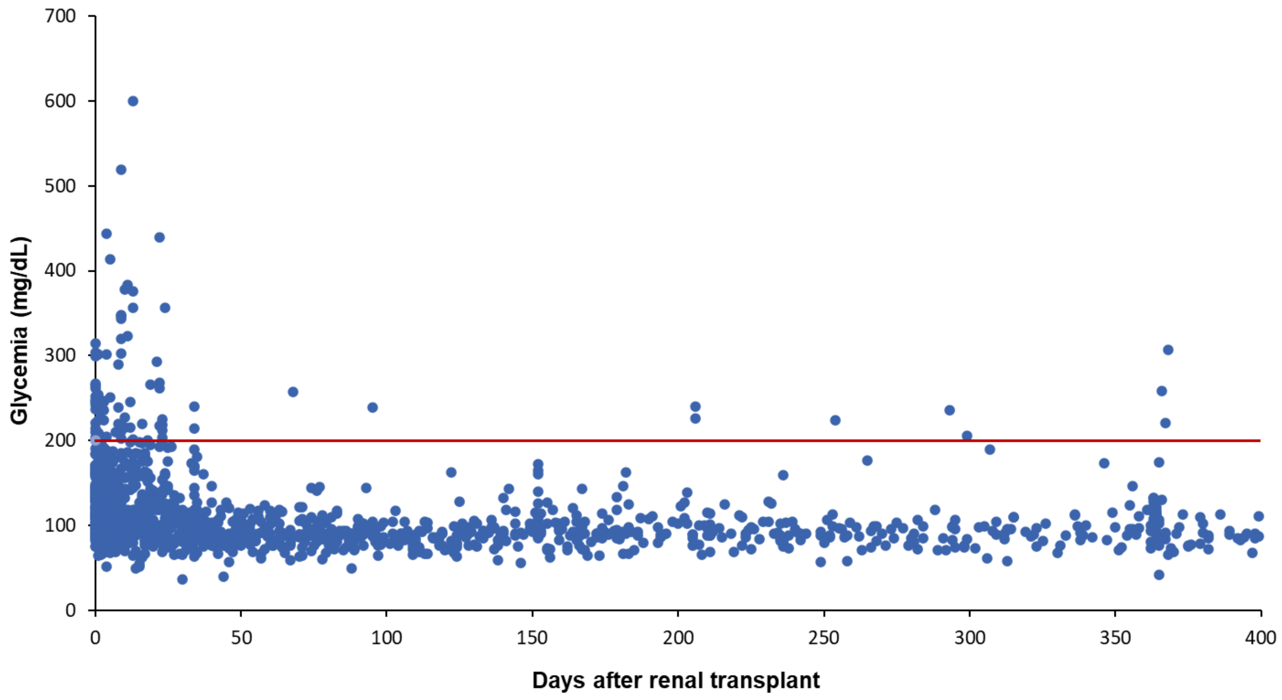

700
600
500
400
300
200
100
0
Glycemia (mg/dL)
0
50
100
150
200
250
300
350
400
Days after renal transplant
